# Thoracolumbar epidural anaesthesia with 0.5% bupivacaine with or without methadone in goats

**DOI:** 10.1186/s13620-017-0093-x

**Published:** 2017-05-26

**Authors:** Priscila dos Santos Silva, Paulo Fantinato-Neto, André Nicolai Elias Silva, Eduardo Harry Birgel Junior, Adriano Bonfim Carregaro

**Affiliations:** 1 Department of Veterinary Science, School of Animal Science and Food Engineering, University of São Paulo, 225th, Duque de Caxias Norte Avenue, Campus Pirassununga, 13635-000 Pirassununga, SP Brazil; 2 Moura Lacerda University Center, 1520th, Dr. Oscar de Moura Lacerda Avenue, Campus Ribeirão Preto, 14076-510 Ribeirão Preto, SP Brazil

**Keywords:** Epidural anaesthesia, Local anaesthetic, Opioid, Ruminant

## Abstract

**Background:**

Epidural anaesthesia is one of the most commonly used locoregional techniques in ruminants. The lumbosacral epidural technique is reasonably easy to perform and requires low volumes of local anaesthetic drug to allow procedures caudal to the umbilicus. However, surgical procedures in the flank of the animal would require an increased volume of drugs. The anaesthetized area provided by thoracic epidural technique is larger than the lumbosacral technique; however the former is rather challenging to perform. Therefore, access through lumbosacral area to introduce a catheter into the thoracolumbar space is a potential alternative to thoracic access. Epidural anaesthesia is achieved with local anaesthetics; opioids can be added to improve analgesia. This study aimed to evaluate the effects of 0.5% bupivacaine with or without methadone, administered through an epidural catheter inserted through the lumbosacral access and advanced to the thoracolumbar space, on thoracolumbar epidural anaesthesia in goats.

**Methods:**

Six animals received two treatments each in a randomized crossover study: BUP treatment consisted of 0.5% bupivacaine (1 mL per each 10 cm of spine column; 1 ± 0.2 mg/kg BW) and BMT treatment was the same; however 1 mL of bupivacaine was replaced by 1 mL (0.22 ± 0.03 mg/kg BW) of methadone (10 mg/mL). The treatments were administered near to T11-T12 through an epidural catheter. Motor blockade and analgesia were evaluated by electrical stimulation.

**Results:**

Heart rate, respiratory rate, ruminal motility and rectal temperature were evaluated before and after the treatment. Motor blockade was observed on both treatments, up to 6 h post-treatment. Analgesia was observed on BUP up to 4 h and on BMT up to 6 h post-treatment. Physiological values did not change at any moment.

**Conclusions:**

Bupivacaine-methadone combination promoted longer-lasting analgesia in goats compared to bupivacaine alone when administered through an epidural catheter into the thoracolumbar space.

**Electronic supplementary material:**

The online version of this article (doi:10.1186/s13620-017-0093-x) contains supplementary material, which is available to authorized users.

## Background

Epidural anaesthesia is one of the most widely used techniques in ruminant surgery. In addition to being inexpensive, its routine feasibility, the specific regional blockade and rapid recovery of the animal are advantages of this technique over others [1, 2].

Epidural anaesthesia, achieved with epidural injection of a local anaesthetic drug, can be applied at any point along the animal’s spine [3]; one of the most common areas is the lumbosacral region [4]. This access promotes dose-dependent anaesthesia over a potentially wide region, from innervations caudal to the diaphragm to the pelvic limbs (reviewed by Galatos [5]), enabling surgical procedures performed through the flank area of the animal (Skarda [6]; reviewed in Plummer and Schleining [4]). However, it causes motor blockade.

Epidural analgesia, achieved with the epidural injection of an analgesic drug, such as an opioid agonist, is a widely used technique in veterinary and human medicine in order to provide analgesia without motor blockade in patients during the trans and post-operative periods [7–10]. Although epidural anaesthesia and analgesia in ruminants is most commonly achieved through a lumbosacral or sacrococcygeal injection, thoracolumbar technique has also been described [11]. Unlike lumbosacral epidural anaesthesia, the blocked area in thoracolumbar anaesthesia can reach from C6 to L4 depending on the volume of anaesthetic used, without motor blockade of the pelvic limbs, thus favouring abdominal surgery such as laparotomy and splenectomy [11, 12].

Bupivacaine is a long-acting local anaesthetic agent that is used in small ruminant surgery when prolonged blockade is needed. Like other local anaesthetics in the epidural space, bupivacaine can affect physiological parameters causing bloating, sleepiness and tremors, requiring more rigorous monitoring when used in ruminants [10]. However, it has not shown to exhibit negative effects on biochemical and haematological parameters and blood gases [13].

In small animals, the analgesic action of epidurally administered morphine is well recognized in pain control, promoting animal well-being and long-lasting analgesia [14]. In sheep, thoracic epidural administration of bupivacaine and morphine in combination demonstrated prolonged analgesia with a lower dose of both drugs when compared to bupivacaine or morphine alone [11]; however the same authors failed to show similar effect when bupivacaine was combined with methadone and administered via the lumbosacral route [15].

Morphine and methadone are safe when administrated by epidural route: no differences were observed in heart rate, blood pressure or respiratory rate in sheep after epidural administration [11, 15].

This study aimed to evaluate the anaesthetic and analgesic effects of bupivacaine alone or in combination with methadone on thoracolumbar epidural anaesthesia in goats.

## Methods

All the following procedures were conducted according to the National Council of Animal Control and Experimentation (CONCEA - Law 11794/08) and approved by the Institutional Animal Care Committee (n. 5186310315).

Six female goats aged between 3 and 4 years and weighing 46.4 ± 6.3 kg were used. All the animals were considered healthy based on physical (heart rate, respiratory rate, mucous membrane color, capillary refill time and ruminal movement) and coproparasitological examinations and laboratory tests (complete blood cell count and liver and kidney screening – alkaline phosphatase, aspartate aminotransferase, bilirubin, gamma-glutamyltransferase, urea and creatinine).

Each animal received two treatments, namely: epidural administration of 0.5% bupivacaine with epinephrine (0.5% Neocaine®, Cristalia Produtos Quim. Farm. Ltda, Itapira, São Paulo, Brazil) at a volume of 1 mL/10 cm of spine length (1 ± 0.2 mg/kg BW) (BUP treatment or BUP), and the same treatment, however 1 mL of bupivacaine was replaced by 1 mL (0.22 ± 0.03 mg/kg BW) of methadone (10 mg/mL) (Mytedom®, Cristalia Produtos Quim. Farm. Ltda, Itapira, São Paulo, Brazil) (BMT). Spine length was measured between the atlanto-occipital and sacrococcygeal joints. The randomization was performed using an internet platform (http://www.randomization.com). A minimum interval of 3 days was maintained between treatments. The observers were unaware of the treatment assignments.

On the day before the first treatment the animals were instrumented. For that, the animals were sedated with 0.1 mg/kg BW of xylazine intramuscularly (Rompun®, Bayer S.A., São Paulo, São Paulo, Brazil) prior to hair clipping and antiseptic preparation of the lumbosacral region. Then, 1 mL of 2% lidocaine (Xylestesin^TM^, Cristalia Produtos Quím. Farm. Ltda, Itapira, São Paulo, Brazil) was administrated subcutaneously at the puncture site. The animals were placed in right lateral recumbency and a 14G Tuohy needle was inserted into the lumbosacral epidural space, as confirmed by the hanging drop test. A 16G epidural catheter (Portex Minipack Epidural Catheter, Smiths Medical International Ltd, Ashford, Kent, UK) was introduced cranially near to the T11-T12 space, which was confirmed by radiographic examination after administration of 2.5 mL of iohexol as contrast (Omnipaque 300, Farmasa, São Paulo, São Paulo, Brazil). After confirmation, the catheter was fixed to the lumbosacral region with a sterile adhesive barrier (Tegaderm™Film, 3 M Health Care, Ontario, Canada) and 0.1 mg/kg BW of 1% yohimbine (Ioimbina 1%, Kaja Vet Farmácia Veterinária, São José do Rio Preto, São Paulo, Brazil) was administrated intravenously (IV) to reverse the effects of xylazine, in order to obtain a faster recovery from sedation. At the end of the procedure, an Elizabethan collar was used in each animal and they were kept in stalls with *ad libitum* access to food and water.

On the next day, noxious stimuli were applied to the left flank region, using a pair of subcutaneous needles connected to an electrical stimulator (Medcir® MT-104, São Paulo, São Paulo, Brazil) with a 50 Hz frequency and 10 to 65 mA current. During application of the stimuli, the following variables were assessed: Response to stimulation (yes or no), degree of ataxia (score 0 to 2) and analgesia (score 1 to 4) (Table 1). In case of negative response to the minimal stimulus (10 mA), successive stimuli (with 1-min intervals) were applied (20, 30 and 65 mA respectively) until a positive or negative response was obtained. Once the animals had a positive response to a lower stimulus, there was no need in applying a higher stimulus. The stimulus was applied before treatment (baseline – 0 min), at 15, 30, 60 and every 60 min thereafter until the animal’s response was the same as at the baseline. Stimuli at the same frequency and current used for each animal were applied on the skin of the left forearm, at radial region, which was used as positive control.

**Table 1:** Scale for assessment of response to noxious stimulus to the left flank, ataxia and analgesia of six goats that received thoracolumbar epidural anaesthesia

Score	Description
Response to noxious stimulus	
No	No response to noxious stimulus or mild response with panniculus reflex
Yes	Response to electrical stimulation, evident panniculus reflex and movement of head and/or tail
Ataxia	
0	No ataxia
1	Difficulty to move, but manages to remain standing without assistance
2	Recumbency
Analgesia	
1	Normal response, with a vigorous and fast reaction to noxious stimulus
2	Decreased response, but with tail movement in response to noxious stimulus
3	Moderate response to noxious stimulus, but animal is restless
4	Full analgesia, animal is quiet and indifferent to noxious stimulus

Heart rate (HR), measured by auscultation of the heart, respiratory rate (f_R_) by auscultation of lung fields, rectal temperature (T) by a digital thermometer, and ruminal movements (RM) by auscultation of the left flank were assessed immediately before electrical stimuli. In addition, 1 mL of blood was collected from the auricular artery at baseline and at 60 min after treatment to assess potential of hydrogen (pH), arterial partial pressure of oxygen (PaO_2_), arterial partial pressure of carbon dioxide (PaCO_2_), arterial oxygen saturation (SaO_2_), bicarbonate concentration (HCO_3_^−^) and base excess, using a blood gas analyzer (iStat1®, Abbott, Chicago, Illinois USA).

The quantitative variables were submitted to normality analysis by the Kolmogorov-Smirnov test (Prism, GraphPad Software, California, USA). Analysis of variance (ANOVA) for paired samples and the Bonferroni’s test for mean comparisons within each treatment relative to baseline were used. Student’s *t* test was used for intertreatment comparisons for HR, f_R_, T and RM. Response to noxious stimuli, ataxia and analgesia were analyzed by Friedman’s nonparametric test. Physiological variables were expressed as mean ± standard deviation (SD) and the other variables were expressed as median ± interquartile range (IR). The differences were considered significant when *P* < 0.05.

## Results

The sedation protocol used for catheter placement together with the recumbent position adopted allowed the epidural catheter to be introduced easily and safely, facilitating the correct positioning of the epidural catheter into the epidural space, between T11 and T12, as confirmed by radiography. Importantly, the catheter remained in the correct position until the end of the experiment in all the animals subjected to the procedure.

**Fig. 1:**
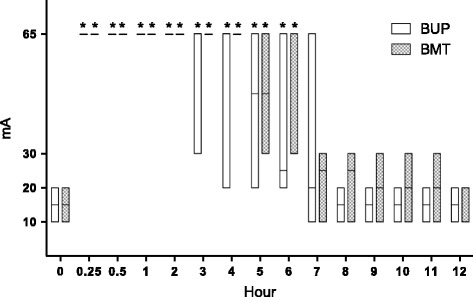
Positive response to noxious stimuli in goats that received thoracolumbar epidural anaesthesia with bupivacaine (BUP treatment) or bupivacaine plus methadone (BMT treatment). *Significantly (*P* < 0.05) different from baseline

At baseline (0 min), six animals showed a positive response to the electrical stimulus at a current of 10 mA (three from BUP treatment and three from the BMT treatment) and another six animals showed positive response at a current of 20 mA (three from BUP and three from BMT). The response was considered positive when the animal exhibited skin twitch and tail and/or head movements. Fifteen minutes later, all the animals from both treatments showed complete local anaesthetic blockade, and did not respond to electrical stimulus at a current of 65 mA. This response was uniform in both treatments until 2 h post-treatment. Some of BUP animals started to respond to sub-maximal electrical stimuli after 3 h, with the same effect occurring in BMT treatment only after 5 h. The response to the noxious stimuli was different from baseline in both treatments, up to 6 h post-treatment. Even though animals treated with BUP showed faster recovery than those treated with BMT, there was no statistical difference between treatments for recovery time (Fig. 1).

**Fig. 2:**
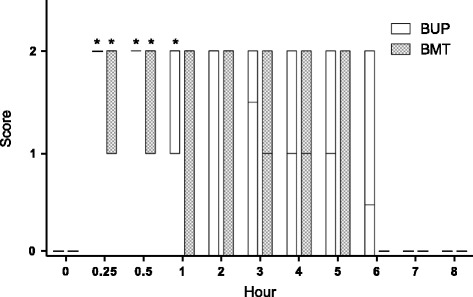
Ataxia scores in goats that received thoracolumbar epidural anaesthesia with bupivacaine (BUP treatment) or bupivacaine plus methadone (BMT treatment). *Significantly (*P* < 0.05) different from baseline

When assessing ataxia, no animal exhibited any abnormality at baseline. At 15 min, all BUP animals already had an ataxia score of 2, remaining recumbent. This treatment showed statistical difference compared to baseline, between 15 and 60 min of assessment. At two hours post anaesthetic administration the animals started to exhibit lower scores, and all animals in BUP treatment received an ataxia score of 0 (no ataxia) after 7 h. In BMT treatment four goats received an ataxia score of 2 and two others a score of 1 after 15 min; these two goats had difficulty moving but managed to remain standing without help. The ataxia in these animals lasted for 30 min. None of the BMT animals presented ataxia from 6 h onwards (Fig. 2).

**Fig. 3:**
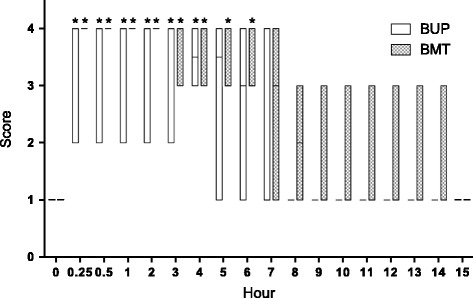
Analgesia scores in goats that received thoracolumbar epidural anaesthesia with bupivacaine (BUP treatment) or bupivacaine plus methadone (BMT treatment). *Significantly (*P* < 0.05) different from baseline

Regarding analgesia, all animals received an analgesia score of 1 (no analgesia) at baseline, showing an intense response and fast reaction to the noxious stimulus. At 15 min, all animals from BMT treatment and five out of six animals from BUP treatment had an analgesia score of 4. Compared to the baseline, BUP treatment showed a statistically different analgesia score up to 4 h post-epidural, whereas in animals in BMT treatment such difference persisted for up to 6 h. However, we would like to emphasize that four animals from BMT treatment received higher analgesia scores compared to baseline for up to 7 h, and in two of them the score remained higher than baseline (even though it was not statistically significant) for up to 11 h post-treatment (Fig. 3).

In the assessment of physiological variables, neither treatment showed significant differences in relation to any of the parameters when compared to baseline, or between each other (Table 2).

**Table 2:** Physiological values (mean ± SD) of goats that underwent thoracolumbar epidural anesthesia with bupivacaine (BUP) and bupivacaine plus methadone (BMT)

		Time (min)				
Parameter	Group	0	15	30	60	120
HR	BUP	82 ± 10	94 ± 17	94 ± 10	92 ± 10	98 ± 18
	BMT	108 ± 20	99 ± 16	91 ± 15	95 ± 16	95 ± 13
f_R_	BUP	28 ± 12	24 ± 5	22 ± 3	23 ± 2	25 ± 8
	BMT	36 ± 17	22 ± 6	24 ± 10	21 ± 8	23 ± 8
RM	BUP	2 ± 1	3 ± 1	2 ± 1	2 ± 1	2 ± 1
	BMT	2 ± 0	2 ± 1	2 ± 1	2 ± 0	2 ± 0
T	BUP	38.7 ± 0.4	38.5 ± 0.3	38.6 ± 0.5	38.6 ± 0.6	38.6 ± 0.6
	BMT	38.7 ± 0.5	38.6 ± 0.4	38.7 ± 0.3	38.6 ± 0.5	38.7 ± 0.6
pH	BUP	7.46 ± 0.06	-	-	7.46 ± 0.04	-
	BMT	7.46 ± 0.02	-	-	7.47 ± 0.02	-
PaO_2_	BUP	79 ± 20.2	-	-	83 ± 15.9	-
	BMT	65 ± 26.2		-	86 ± 8.7	-
PaCO_2_	BUP	34 ± 5.1	-	-	33 ± 5.7	-
	BMT	32 ± 3.7	-	-	31 ± 3.6	-
HCO_3_^−^	BUP	24.7 ± 5.1	-	-	23.7 ± 4.3	-
	BMT	22.6 ± 2.6	-	-	22.8 ± 2.4	-
SaO_2_	BUP	93 ± 7.3	-	-	95.4 ± 2.6	-
	BMT	95 ± 2.2	-	-	96.4 ± 1.3	-
BE	BUP	1.4 ± 5.6	-	-	0.4 ± 4.8	-
	BMT	−1 ± 2.6	-	-	−0.5 ± 2.1	-

*HR* heart rate (beats/min), *f*_*R*_ respiratory rate (mov/min), *RM* ruminal movements (mov/min), *T* temperature (^o^C), *pH* potential hydrogen, *PaO*_*2*_ arterial oxygen tension (mmHg), *PaCO*_*2*_ arterial carbon dioxide tension (mmHg), *HCO*_*3*_^*−*^ bicarbonate (mmol/L), *SaO*_*2*_ arterial oxygen saturation (%), *BE* base excess (mmol/L)

## Discussion

The choice of drugs used in anaesthesia of ruminants is closely related to both recovery from anaesthesia and the undesirable effects of these drugs (Reviewed by Galatos [5]). We chose bupivacaine as it is one of the most commonly used local anaesthetics in both human [16] and veterinary medicine [8, 17–20], and combined it with methadone because the combination of bupivacaine and opioids has demonstrated satisfactory results in pain management in ruminants [11, 15]. The thoracolumbar epidural route was chosen in this study in an attempt to decrease the total volume administered and to promote better anaesthesia compared to local infiltration techniques. Furthermore, we chose the thoracic epidural anaesthetic technique due to the blockade it provides, which extends from the emerging branches of T13 to the pelvis [2], thus providing satisfactory analgesia of the flank region without recumbency, which was one of the objects of the present study. Other epidural anaesthesia techniques, such as lumbosacral or sacrococcygeal, block a smaller area of the animal compared to the thoracolumbar technique [4].

The latency period reached in both treatments, up to 15 min, was similar to that observed in uraemic or healthy goats when thoracic epidural bupivacaine was administered [13]. The same period was also observed in sheep submitted to epidural anaesthesia with bupivacaine and methadone [15].

Electrical stimulus is used to measure analgesic efficacy of the local anaesthetics used, and animal behavioural response may be used as a supplementary parameter of anaesthetic blockade efficiency [21]. Even though the anaesthetic blockade started at the same time in both treatments (at 15 min), the BMT treatment promoted longer-lasting analgesia.

Differently, it was reported a shorter duration of analgesia using the combination of bupivacaine and methadone in sheep [15]. This difference might be explained by the higher doses use here, for bupivacaine (1 mg/kg versus 0.25 mg/kg BW) and for methadone (0.2 mg/kg versus 0.15 mg/kg BW).

When administered by the epidural route, methadone can reach systemic absorption, due to its lipophilic characteristic, and therefore it may promote physiological changes (Reviewed by Bujedo et al., [22]). Despite that, in this study we observed no changes in heart rate, respiratory rate, temperature and ruminal movements. Our results corroborate another study, which observed the same pattern, when assessing the influence of methadone injected IV into healthy sheep [15]. Similar results were obtained in sheep that received both bupivacaine alone and bupivacaine combined with morphine by thoracic epidural administration [11]. Runa et al. [23] observed significant differences in some physiological parameters in goats that received bupivacaine by the epidural route [23], probably due to the age of the animals and the dose used.

It is important to note that the complications attributed to epidural anaesthesia are considered rare [24]. Nevertheless, some authors reported a negative influence of bupivacaine alone and in combination with morphine in the PaCO_2_ and PaO_2_ of sheep, with no significant differences in pH and SaO_2_ [11]. In the present study, no significant changes were observed in blood gas analysis, in line with other studies with bupivacaine in goats [13]. However, one goat in the present study (BUP treatment) showed bloating, muscle twitching and respiratory distress at 15 min, similar to the observations by other authors who reported bloating, muscle twitching and sleepiness in sheep that received bupivacaine by the caudal epidural route [10]. Nonetheless, these effects were not observed in another study with goats [13]. Herein we hypothesize that bloating may be associated with the lateral recumbency, which the animal assumed during the procedure due to the induced motor blockade.

As stated before, in some situations it would be ideal to provide local anaesthesia without motor blockade in the pelvic limbs; however all animals in this study exhibited motor blockade. De Rossi et al. [25] reported that animals that received subarachnoid injection of bupivacaine recovered faster from motor blockade than those receiving lidocaine, in addition to exhibiting a long-lasting analgesia period [25]. In other study, De Rossi et al. [15] reported long-lasting analgesia and ataxia in sheep, when epidural bupivacaine or methadone were administered alone in comparison to the epidural administration of both drugs combined [15]. The extension of motor blockade obtained in the present study may be related to the dose of bupivacaine. The use of lower doses of bupivacaine may avoid motor blockade of pelvic limbs in goats.

## Conclusion

The advantage of the thoracolumbar epidural technique is that it may promote satisfactory analgesia in the animal’s flank, and a larger anaesthetized area. The combination of 0.5% bupivacaine and methadone was more effective in promoting analgesia, with a short-lasting ataxic period and no undesirable effects when compared to bupivacaine alone.

## Additional files

Additional file 1: X-ray showing the correct position of the catheter into the thoracolumbar space (red arrow). (JPEG 1397 kb)

Additional file 2: A pair of subcutaneous needles, positioned on the goat's left flank, through which noxious stimulus was applied. (JPG 2084 kb)

Additional file 3: A pair of subcutaneous needles, positioned on the radial region, which was used as positive control. (JPG 1137 kb)
