# Supplementary figures and images for: Thoracolumbar epidural anaesthesia with 0.5% bupivacaine with or without methadone in goats
Source: Ir Vet J. 2017 May 26;70:15. doi: 10.1186/s13620-017-0093-x (PMC5446691; doi:10.1186/s13620-017-0093-x)

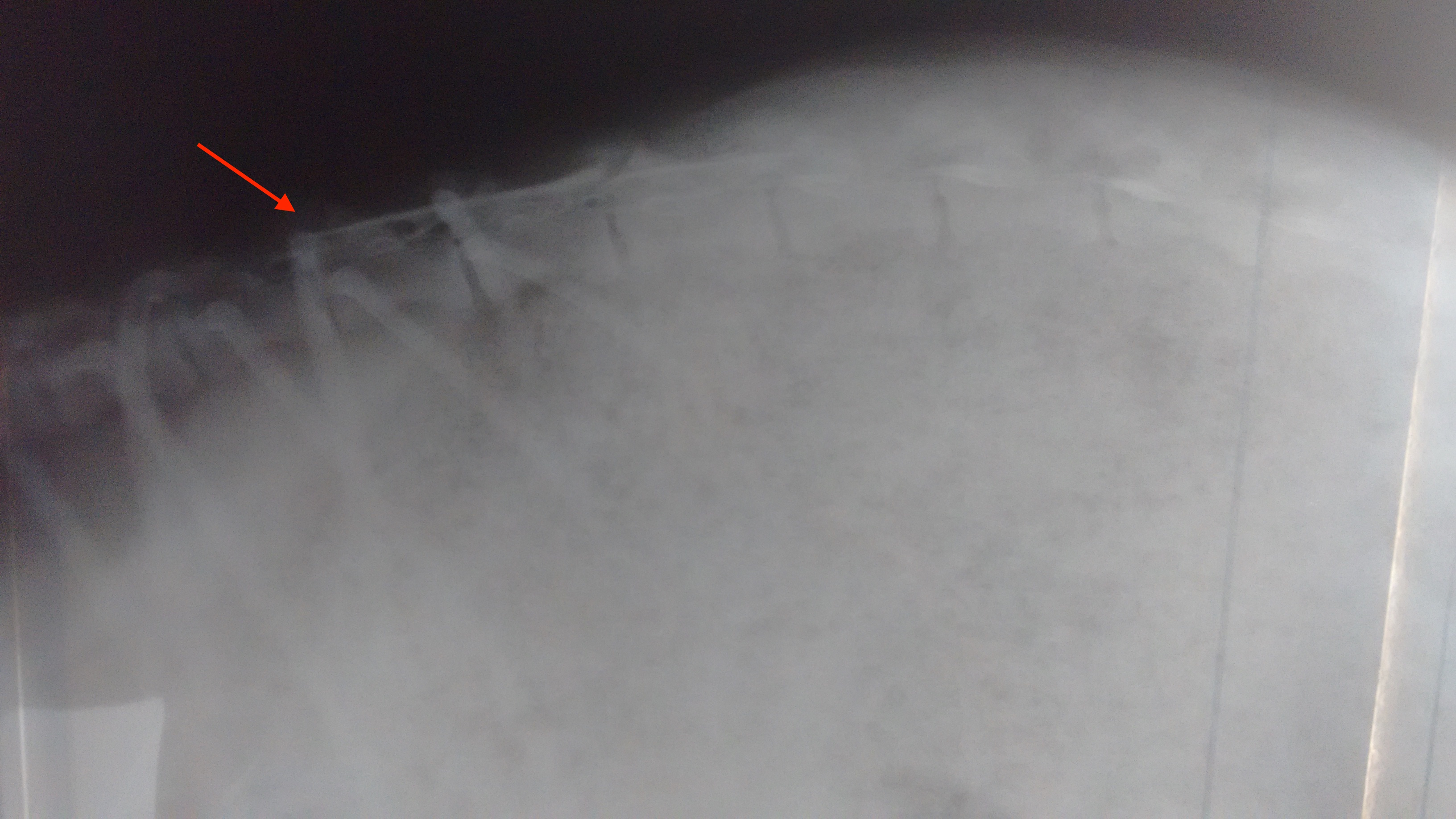

Supplement: Supplementary file 1 — X-ray showing the correct position of the catheter into the thoracolumbar space (red arrow). (JPEG 1397 kb) [file 13620_2017_93_MOESM1_ESM.jpeg]

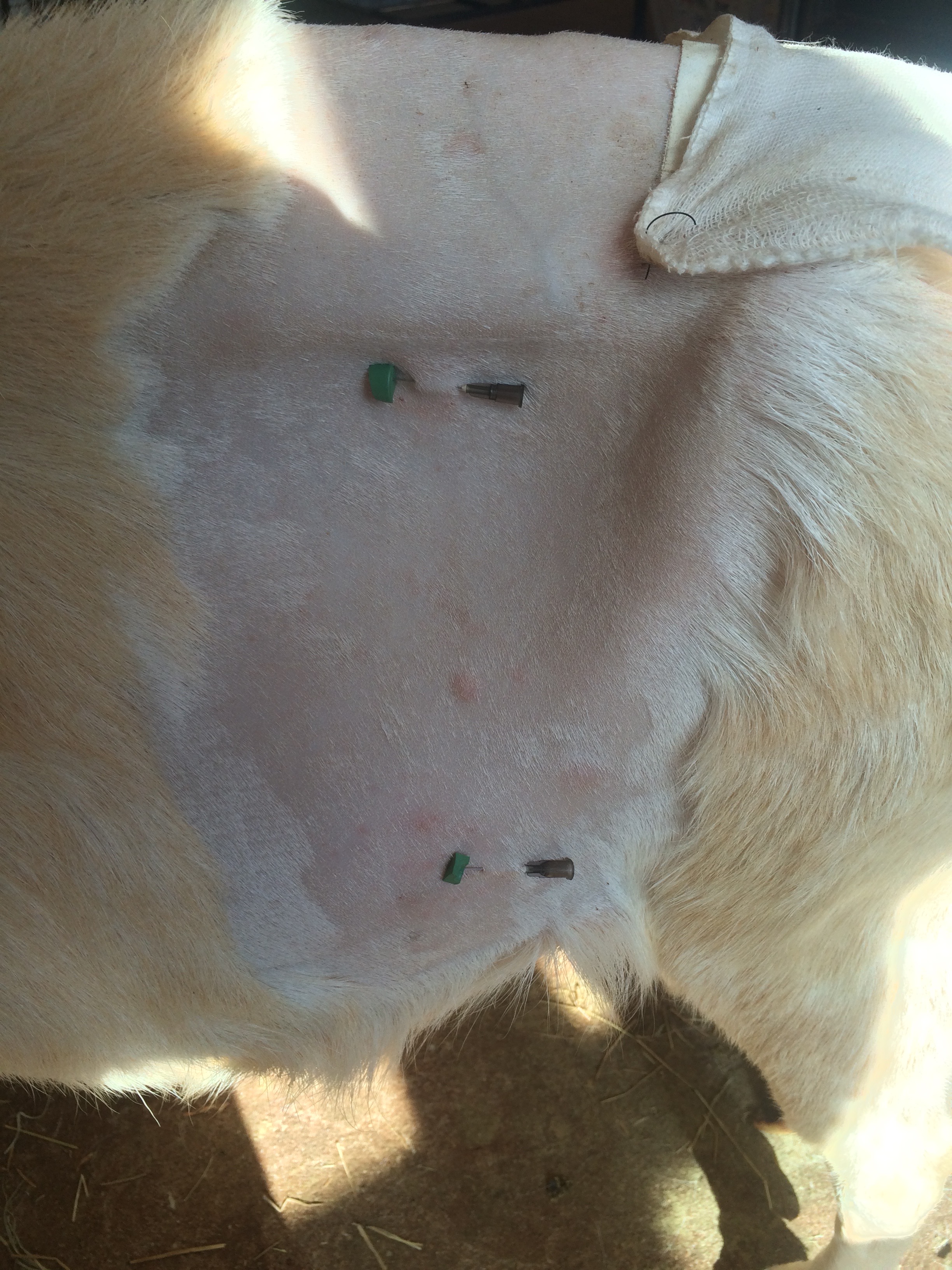

Supplement: Supplementary file 2 — A pair of subcutaneous needles, positioned on the goat's left flank, through which noxious stimulus was applied. (JPG 2084 kb) [file 13620_2017_93_MOESM2_ESM.jpg]

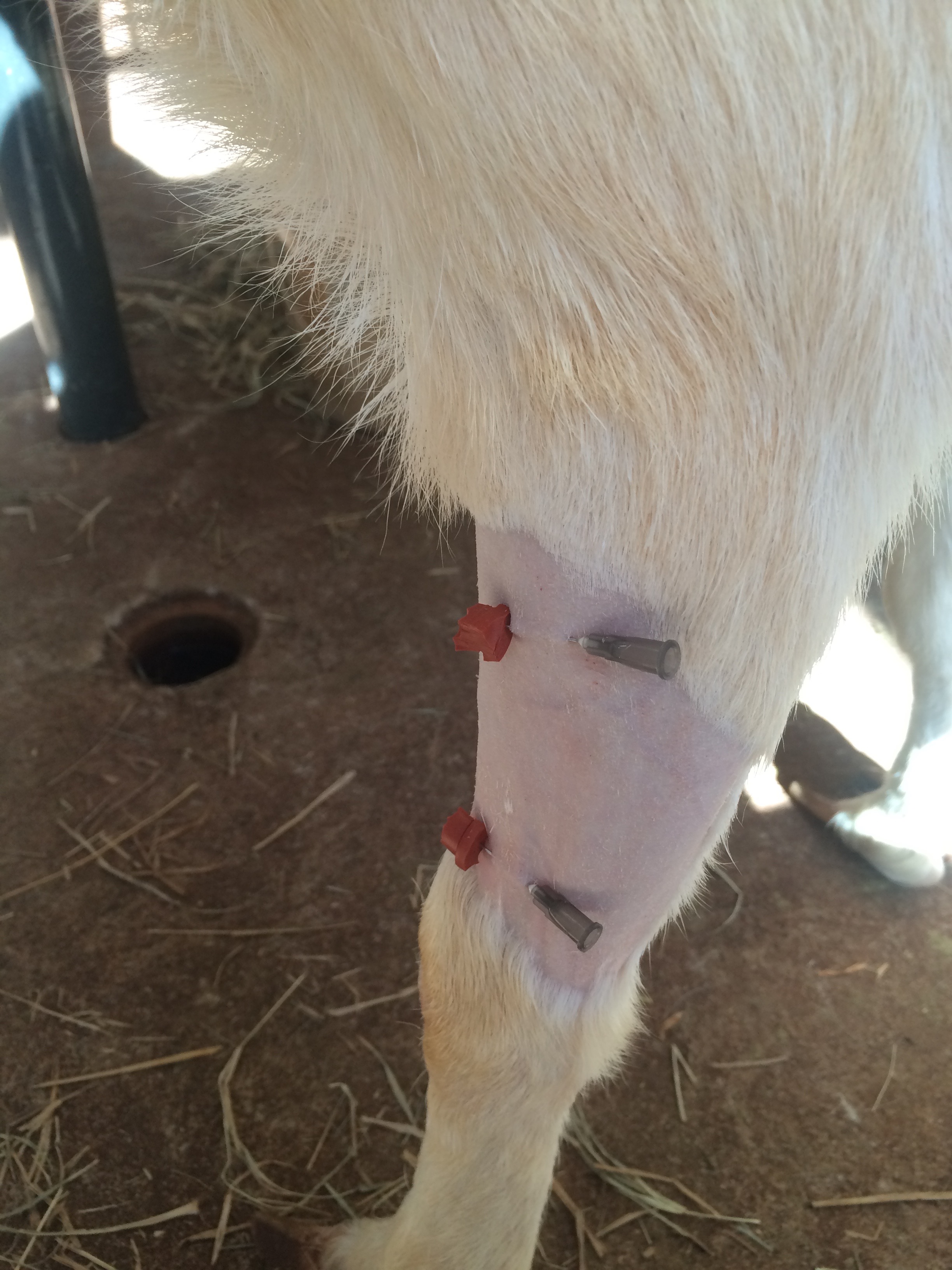

Supplement: Supplementary file 3 — A pair of subcutaneous needles, positioned on the radial region, which was used as positive control. (JPG 1137 kb) [file 13620_2017_93_MOESM3_ESM.jpg]
